# Biobased Composites from Biobased-Polyethylene and Barley Thermomechanical Fibers: Micromechanics of Composites

**DOI:** 10.3390/ma12244182

**Published:** 2019-12-12

**Authors:** Ferran Serra-Parareda, Quim Tarrés, Marc Delgado-Aguilar, Francesc X. Espinach, Pere Mutjé, Fabiola Vilaseca

**Affiliations:** 1 LEPAMAP Group, Department of Chemical Engineering, University of Girona, 17003 Girona, Spain; ferran.serra@udg.edu (F.S.-P.); joaquimagusti.tarres@udg.edu (Q.T.); m.delgado@udg.edu (M.D.-A.); pere.mutje@udg.edu (P.M.); 2 Chair on Sustainable Industrial Processes, University of Girona, 17003 Girona, Spain; 3 Design, Development and Product Innovation, Dept. of Organization, Business, University of Girona, 17003 Girona, Spain; francisco.espinach@udg.edu; 4 Department of Fibre and Polymer Technology, KTH Royal Institute of Technology, SE-10044 Stockholm, Sweden

**Keywords:** bio-polyethylene, barley straw, thermomechanical fibers, interface

## Abstract

The cultivation of cereals like rye, barley, oats, or wheat generates large quantities of agroforestry residues, which reaches values of around 2066 million metric tons/year. Barley straw alone represents 53%. In this work, barley straw is recommended for the production of composite materials in order to add value to this agricultural waste. First of all, thermomechanical (TMP) fibers from barley straw are produced and later used to reinforce bio-polyethylene (BioPE) matrix. TMP barley fibers were chemically and morphologically characterized. Later, composites with optimal amounts of coupling agent and fiber content ranging from 15 to 45 wt % were prepared. The mechanical results showed the strengthening and stiffening capacity of the TMP barley fibers. Finally, a micromechanical analysis is applied to evaluate the quality of the interface and to distinguish how the interface and the fiber morphology contributes to the final properties of these composite materials.

## 1. Introduction

The production of environmentally friendly materials is a key point to more sustainable development. Today’s society has become aware of the need to eliminate its dependence on fossil resources. In this sense, the production of materials coming from renewable resources with properties similar to the current ones is becoming a necessity [1,2,3,4,5,6]. There are two main ways to develop greener plastic materials—bio-based and biodegradable or bio-based and recyclable materials [5,7,8,9,10]. Both types must coexist in order to achieve the properties currently provided by materials manufactured from fossil origin resources. 

Biopolyethylene is produced from biomass such as sugarcane [11] and is an alternative to polyethylene obtained from oil. However, due to market dimensions and scale economies, the cost and price of bio-based polymers are higher than common plastics. Hopefully, a higher penetration in the market of bio-based materials will reduce its production costs and then its price [12].

In the field of bio-based and recyclable materials, the use of bio-polyethylene reinforced with natural fibers is of great interest. By combining a bio-based matrix such as biopolyethylene with a bio-based fiber reinforcement, it is possible to obtain high performance and recyclable bio-composites [13]. Moreover, if an agroforestry residue is suggested as raw material, a new renewable, cheap, and sustainable reinforcement is used, thus adding value and extending the agroforestry value chain. Barley straw has a world production of 195,000,000 bdmt/year, representing 15.6% of cereal straws world-wide [14]. However, to the best of our knowledge, there are very few publications on the use of this residue as raw material, and none in the field of composite materials [15,16,17,18].

The use of natural fibers as reinforcements in plastic matrix composites has a number of challenges. The main ones are the poor interaction at fiber-matrix interface and the low degradation temperatures of natural fibers. The former is related to the incompatibility between highly hydrophilic fibers and the hydrophobic polymer matrices. Under these circumstances, there is not a good interaction between the matrix and the reinforcement, and a strong interface is impossible to attain [19]. This polarity difference can be overcome by adding coupling agents. It is already known in the literature that coupling agents such as maleated polyethylene (MAPE) can improve the strength of the interface between the two phases [20,21]. However, this interaction must be studied in each case because the surface chemical composition of the fibers is also a key factor [22]. On the other hand, the degradation temperature of the cellulosic fibers limits the processing temperatures, which have to be kept below 220 °C [23].

In this work, the production of composite materials of thermo-mechanical barley fibers with bio-polyethylene is studied. Composite materials with MAPE contents ranging from 0 to 8 wt % are produced to elucidate the optimal coupling agent in the formulation that renders the highest tensile strengths. From here, coupled composites from 15–45 wt % of thermo-mechanical barley fibers are obtained, and their mechanical properties are analyzed and discussed. A micromechanics analysis of the tensile strength is carried out in order to assess the strength of the interface and the intrinsic tensile strength of the reinforcements.

## 2. Materials and Methods 

### 2.1. Materials

Polyethylene based on renewable sources kindly supplied by Braskem (São Paulo, Brazil) was used as polymer matrix. This 100% recyclable biopolyethylene (BioPE) is obtained from sugarcane and therefore is bio-based. The polymer has a molecular weight of 61.9 g/mol, a melt flow index of 20 g/10 min at 190 °C with 2.16 kg, and a density of 0.955 g/cm^3^. Maleic anhydride-grafted-polyethylene (MAPE) with a maleic–anhydride substitution of 0.9% (Fusabond MB100D) was provided by DuPont (Wilmington, DE, USA). 

Barley straws were kindly provided by Mas Clarà S.A. (Girona, Spain). 

### 2.2. Methods

Initially, the barley straw was chopped by means of a blade mill equipped with a 3 mm mesh. Then, the barley straw was subjected to a thermo-mechanical digestion process. This process was carried out in a pressure reactor at 160 °C and a liquid ratio of 1:6. The fibers extracted from the reactor were washed repeatedly and then passed through a Sprout–Waldron defibrator. The obtained fibers were filtered and dried at 80 °C.

**Figure 1:**
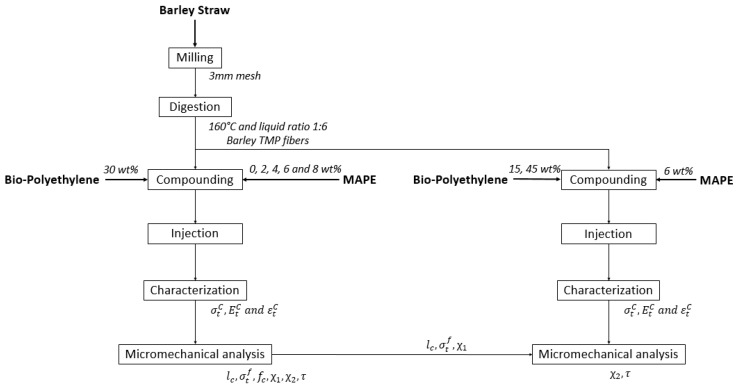
Flow chart of the composites production and characterization.

The study of composite materials produced from bio-polyethylene and thermo-mechanical fibers of barley straw was divided in two parts. In the first step, the amount of coupling agent was optimized for composites comprising 30 wt % of fiber content. From these results, a micromechanics analysis allowed us to obtain the orientation factor and the intrinsic resistance of the barley thermo-mechanical fibers. In the second step, the mechanical properties of composite materials adding different percentages of fibers were studied. The analysis was based on the micromechanical values resulting from the optimization of the coupling agent. A flow chart of the composites production and their characterization is shown in Figure 1.

The compounding process was performed using a Gelimat intensive kinetic mixer (Dusatec, Inc., Ramsey, NJ, USA). The fibers were introduced at a rotor speed of 300 rpm. Subsequently, and maintaining this constant speed, the polymer was incorporated together with the coupling agent. The composites were produced with fiber percentages between 15 and 45 wt %. Once all materials were in the kinetic mixer, the rotor speed was increased up to 3000 rpm. The increase in the rotor speed cause increase in the temperature reaching the melting temperature of the polymer. The material was discharged once the matrix was melted and mixed with the fibers. The blend was then cooled down and subsequently pelletized. The composites were kept in an oven at 80 °C temperature for 24 h before injection molding. The mold injection was carried out using a 220 M 350–90U injection machine (Arburg, Loßburg, Germany). A series of 20 standard test specimens of each composite were mold injected to assess tensile properties.

The morphological study of fibers was carried out using a MORFI equipment (Techpap, Gières, France). The fibers into the composite materials were extracted using a Soxhlet apparatus with Decalin to dissolve the matrix. From here, the mean fiber lengths, fiber diameters as well as fiber length distributions were obtained. The chemical composition of the fibers was determined from the analysis of ash content (ISO 2144:2019 standard [24]), extractives (TAPPI T204 cm-07 [25]), lignin klason (ISO/DIS 21436 [26]), and holocellulose.

Tensile tests were performed according to ASTM D790 standard. All samples were conditioned at 50% of relative humidity and 23 °C in a climatic chamber (Dycometal, Viladecans, Spain) during at least 48 h before testing (ASTM D618 standard [27]). The tensile tests were carried out in an Instron TM 1122 universal testing machine (Instron, Cerdanyola, Spain). This equipment is fitted with a 5 kN load cell. The experimental results are the average of at least of testing five samples.

## 3. Results and Discussion

### 3.1. Fibers Assessment

Table 1 shows the results from the chemical analysis of the barley straws, the fibers from submitting barley straws to a thermomechanical process, and spruce fibers subjected to the same process. It is evident from the table that there are significant differences between the chemical composition of virgin raw material (barley straw) and the fibers after a thermomechanical process.

These differences are due to the thermomechanical treatment. More in detail, the thermal treatment acts on the middle lamella and the primary cell wall of the fibers removing part of the ashes, extractives and lignin, mainly present in these areas of the fiber structure. Thereafter the secondary wall is reached (S1, S2, and S3), where higher contents of cellulose and hemicellulose are present. Afterward, when these fibers are subjected to a mechanical defibration process (Sprout–Waldron defibrator), the fiber agglomerates tend to split at the fiber–fiber union. In comparison to a thermomechanical treatment, an exclusively mechanical process would break the structure of the fibers in a disordered way while preserving the same chemical composition of the raw material. On the other hand, as shown in Table 1, the chemical composition of fibers from spruce wood treated by the same thermomechanical process has a higher lignin content. This is due to the different chemical composition of wood fibers with respect to an agroforestry waste. It has been reported, that fibers from non-wood resources present lower lignin contents [28]. This difference in chemical composition can be related to their surface composition [22,29]. Börås and Gatenholm [22] developed a model for the distribution of carbohydrates, lignin, and extractives on the surface of fibers after defibration. This model proposes that lignin is covering in a heterogeneous way the carbohydrates that are in an ordered phase in the form of fibrils. In this model, it is also proposed that the extractives form globular particles spread all over carbohydrates and lignin. These chemical surface properties are essential to understand the difficulty to achieve composites with strong interfaces between natural fibers and polyolefin matrices.

**Figure 2:**
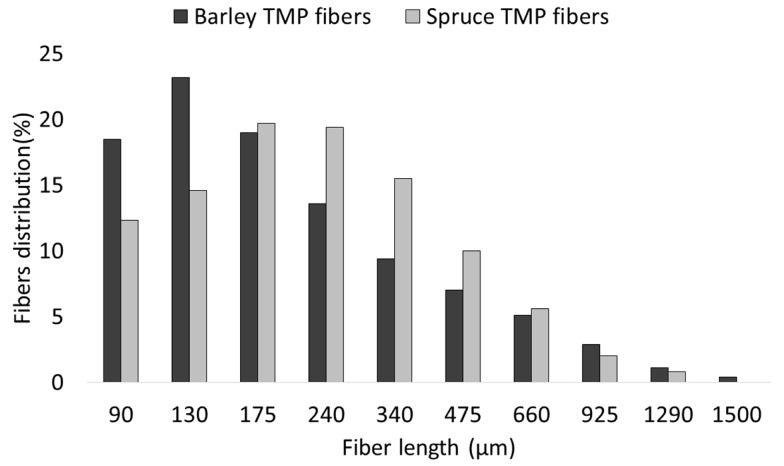
Length distributions of barley TMP and spruce TMP fibers.

Figure 2 shows the length distributions of barley TMP fibers and spruce TMP fibers. The determination of the morphology of the fibers is important to determine their reinforcement capacity. In the same way as chemical composition, wood fibers tend to show higher aspect ratios (length/diameter) than fibers from annual plants [28]. Nonetheless, the morphology of the fibers is remarkably changed during composite processing. Compounding and mold injection processes cause an important reduction of the mean length of the fibers [29].

### 3.2. Coupling Agent Optimization

The tensile properties of a composite material are mainly impacted by the nature of the reinforcement and the matrix, the reinforcement content, dispersion and orientation, its aspect ratio, and largely by the quality of the matrix–reinforcement interface [30,31,32]. The different nature between natural fibers (hydrophilic) and polyethylene (hydrophobic) hinders the achievement of a strong interface [33,34]. Improvement of the interface strength can be achieved by the use of coupling agents such as polyethylene grafted with maleic anhydride (MAPE) [35,36,37].

Table 2 shows the tensile strength ($\sigma_{t}^{C}$), Young’s modulus ($E_{t}^{C}$), strain at break ($\varepsilon_{max}$), and the contribution of the matrix to the tensile strength of the matrix ($\sigma_{t}^{m^{*}}$) for 30 wt % TMP-reinforced BioPE composites coupled with MAPE percentages ranging from 0 to 8%. The density of the fibers inside the material (1.35 g/cm^3^) was determined from the density of the bio-propylene (0.955 g/cm^3^) and the density of the resulting composite material. Therefore, the volume fraction (*V^F^*) of fibers in a 30 wt % composite material is 0.233.

**Figure 3:**
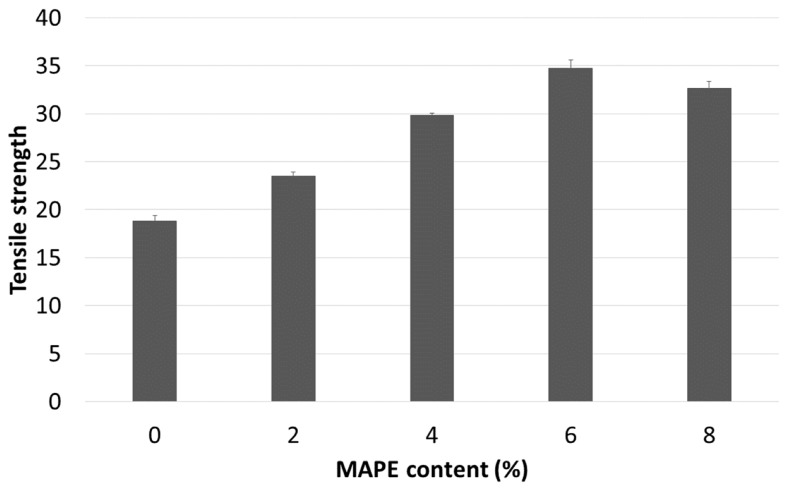
Tensile strength evolution of 30 wt % composite against maleic anhydride (MAPE) content.

The tensile strength of the composites reinforced with 30 wt % of barley TMP composites increased noticeably when MAPE was added in the formulation (Figure 3). However, when the MAPE exceeds 6%, the tensile strength starts to decrease. The increase of the tensile strength of the composites is promoted by the creation of covalent ester bonds. The higher is the MAPE content the higher are the possible bonds. These bonds are formed by the reaction between the anhydride groups of maleic acid and the hydroxyl groups on the surface of the fibers [19,38]. Nonetheless, if a certain percentage of coupling agent is exceeded, grafted polyethylene chains tend to self-entangle and decrease the tensile strength of the composite. In this case, it was resolved that 6% MAPE provides the highest increase in tensile strength of the material (34.70 MPa), which represents an increase over 90% of the tensile strength of the matrix. On the other hand, the Young’s modulus of the composite material is not affected by the quality of the interface. In this sense, composite materials with poor interfaces present Young’s moduli similar to composites with optimized MAPE contents [39]. For the elongation at the break, the deformation capability increases with the higher coupling agent content as a direct consequence of Hooke’s law.

**Figure 4:**
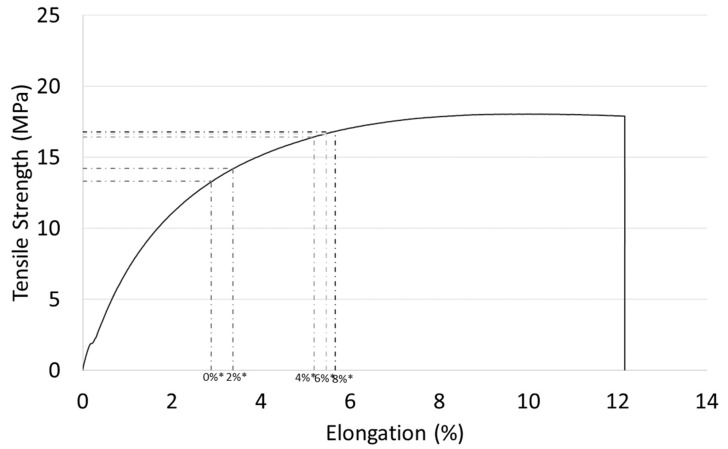
Tensile strength vs elongation diagram of BioPE polymer. Matrix contribution to tensile strength for each MAPE content is also shown.

Once the elongation at break of the composites are known, it is possible to determine the contribution of the matrix to the strength of these material ($\sigma_{t}^{m^{*}}$), as shown in Figure 4. By translating the value of the elongation at rupture of the composites on the stress-strain curve of the matrix, the $\sigma_{t}^{m^{*}}$ value can be obtained.

**Figure 5:**
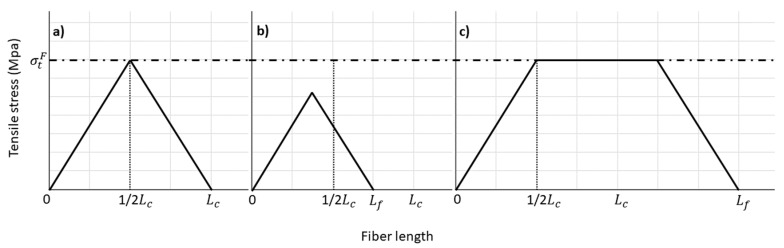
Axial load graphic of (**a**) critical length fibers, (**b**) subcritical length fibers, and (**c**) supercritical length fibers.

Mechanical properties of composites (BioPE + 30 wt % barley TMP fibers) related to coupling agent content were modelled by using a modified rule of mixtures for the tensile strength of semi-aligned short fiber reinforced composites (Equation (1)) [40,41].

(1) $$\sigma_{t}^{C}=f_{c}\cdot \sigma_{t}^{f}\cdot V^{F}+\sigma_{t}^{m^{*}}\cdot \left(1-V^{F}\right),$$

where $\sigma_{t}^{C}$ is the tensile strength of the composite, $\sigma_{t}^{f}$ the intrinsic tensile strength of the fibers and $f_{c}$ a coupling factor. As reported by Sanadi et al. [42], the coupling factor used in the modified rule of mixtures (*f_c_*) is the orientation factor ($\chi_{1}$) times the length and interface factor ($\chi_{2}$) of the fibers inside the composite. Nonetheless, the equation cannot be used to evaluate the intrinsic tensile strength of the reinforcements because has another unknown, the value of the coupling factor. Accordingly, the use of the modified Kelly-Tyson equation (Equation (2)) [43].

(2) $$\sigma_{t}^{C}=\chi_{1}\cdot \left(\sum_{L_{c}}^{i=0}\left[\frac{\tau \cdot l_{i}^{F}\cdot V_{i}^{F}}{d^{F}}\right]+\sum_{\infty }^{j=L_{c}}\left[\sigma_{t}^{f}\cdot V_{j}^{F}\cdot \left(1-\frac{\sigma_{t}^{F}\cdot d^{F}}{4\cdot \tau \cdot l_{j}^{F}}\right)\right]\right)+\sigma_{f}^{m^{*}}\cdot \left(1-V^{F}\right),$$

where *L_c_* is the critical length, *l^f^* the fiber length and *d^f^* the fiber diameter. The Kelly–Tyson equation divides the contribution of the reinforcements to the tensile strength of the composites between the contributions of the subcritical (*l^f^* < *l_c_*) and supercritical (*l^f^* > *l_c_*) fibers. The equation introduces an interfacial shear strength (τ). According to the shear-lag model, the matrix transmits the force from the matrix to the reinforcement by shear forces in the interface. This means that the fibers are fully loaded at the center of their length and nil at their ends. According to the length of each fiber inside the composite, the load in its center will be less or equal to its intrinsic tensile strength (Figure 5).

Therefore, the length of the reinforcements, its intrinsic tensile strength, and the interfacial shear strength command the value of the critical length. Only those reinforcements with lengths equal or higher than the critical will fully deploy its strengthening capabilities. The critical length is equal to the intrinsic tensile strength of the fibers times the fiber radius, divided by the interfacial shear strength.

**Figure 6:**
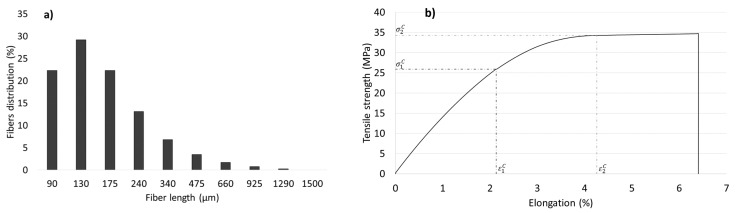
(**a**) Fibers length distribution from 30% composite and (**b**) stress-strain curve of a coupled 30% composite.

The Kelly–Tyson model considers the stress distribution due to a single short fiber embedded in a matrix when the system is subjected to a uniaxial load in the direction of the fiber axis [44]. Nonetheless, Kelly and Tyson’s equation cannot be solved as shows three unknowns, the orientation factor, the critical length and the intrinsic tensile strength of the reinforcements. Anyhow, Bowyer and Bader developed a numerical method capable to solve the equation. The Bowyer–Bader solution [45] assumes that the interfacial shear strength is independent of the deformation, and that the effect of the fiber’s inclination to the load axis can be explained by a scale factor, and the fiber orientation factor. Then, by using the experimental values of two points of the stress-strain curve $\chi_{1}$ and the τ can be determined (Figure 6).

The results of applying the method to a composite that adds 6 % coupling agent returns an orientation factor of 0.309 and an interfacial shear strength of 10.49. The interfacial shear strength obtained is close to von Misses criteria (τ = 10.42 MPa), where $\tau =\sigma_{t}^{m}/\sqrt{3}$ [46]. Von Mises criteria is used to define strong interfaces. Thus, the obtained value can be considered reasonable and corresponding to a strong interface. Then, the critical fiber length was calculated from $l_{c}=\left(d^{f}\cdot \sigma_{t}^{c}\right)/2\cdot \tau$ [47]. The obtained value of critical fiber length was 409.91 μm. Once obtained the values of $\chi_{1}$, τ and $l_{c}$, it was possible to use of Kelly–Tyson equation (Equation (2)) to determine the value of the intrinsic strength of the fibers. The result for a 30% composite with 6% coupling agent was 521.18 MPa. Then, this intrinsic tensile strength was introduced in the modified rule of mixtures (Equation (1)) to evaluate a coupling factor (*f_c_*) for 30% composite that was found to be 0.18. This value is in the range from 0.18 to 0.20 obtained by composites with optimal interfaces [48].

Equation (1) was used, together with the intrinsic tensile strength of the reinforcement, to evaluate the coupling factors for all the composites reinforced with 30 wt % of fibers and MAPE contents ranging from 0 to 8%. The lower value (*f_c_* = 0.07) was obtained for the uncoupled composite.

The following equations show the relation between the coupling factor and the length and interface factor and the orientation factor (Equation (3)). Then, Equations (4) and (5) are used to relate the values of the orientation factor with the interfacial shear strength. Equation (4) is used when the mean length of the reinforcements is higher that the critical length. Otherwise, Equation (5) must be used.

(3) $$f_{c}=\chi_{1}\cdot \chi_{2}$$

(4) $$\tau =\frac{\sigma_{t}^{f}\cdot d^{f}}{4\cdot l^{f}\cdot \left(1-\chi_{2}\right)}\;for\;l^{f}\geq l_{c}$$

(5) $$\tau =\frac{\sigma_{t}^{f}\cdot d^{f}}{l^{f}}\chi_{2}\;for\;l^{f}\leq l_{c}$$

**Figure 7:**
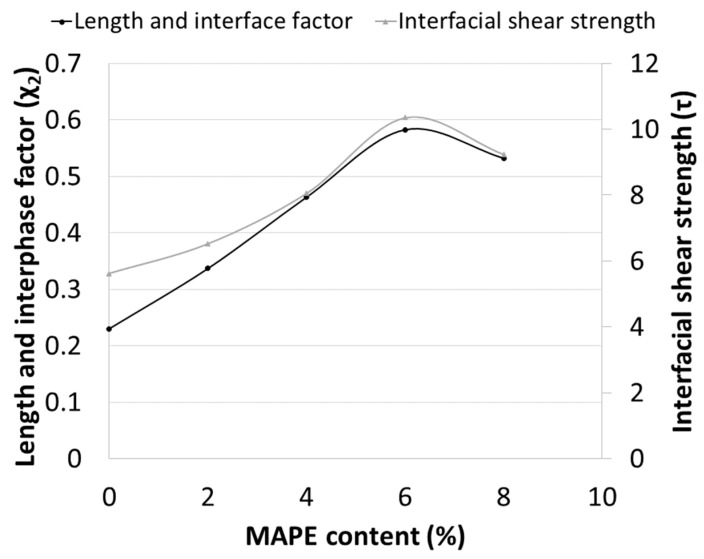
Evolution of the length and interphase factor and the interfacial shear strength as function of coupling agent content.

The orientation factor is affected by the geometry of the injection mold and the mold injection parameters. As long as all the composites were mold injected in the same mold and under the same parameters, is possible to consider that such composites will share the same orientation factor. Thus, the obtained 0.309 orientation factor was used together with the coupling factors and Equation (4) to obtain the length and interface factors of the composites against MAPE content. Likewise, Equations (4) and (5) were used to evaluate the corresponding interfacial shear strength of the same composites. Figure 7 shows these values against MAPE contents. 

The figure shows the evolution of both parameters against MAPE content. In some sense, the length and interface factor integrate the strength of the interface along with the influence of the morphology of the reinforcements. The interfacial shear strength accounts only for the strength of the interface. Thus, both curves are expected to be similar in shape. Major dissimilarities will be caused by the effect of the morphology. Figure 7 shows very similar curves, changing only due to scale effects. Thus, in this case, the main parameter ruling the contribution of the fibers against MAPE content will be the strength of the interface. The effect of the morphology of the fibers will be limited and only due to slight decreases of the mean lengths of the fibers against MAPE content. The figure shows the maximum values for the composite with 6 wt % MAPE contents.

### 3.3. Mechanical Performance of Barley TMP/BioPE Composites

Once the effect of the coupling agent on the tensile strength of the composites was assessed, coupled composites with 15 and 45 wt % reinforcement contents were prepared and tested. Both composites comprise 6% of MAPE, with respect to the fiber content. It can be observed from Table 3 that the increase in the percentage of barley TMP fibers led to a notable increase of the tensile strength, reaching a value of 43.1 MPa for the 45 wt % formulation which represents a 138% increase over the matrix. This increase was slightly higher than those obtained in the bibliography where an addition of 30% natural fibers (maize fibers or corn stalk fibers) does not exceed the 85% increase in tensile strength. This indicates the correct level of interface between fiber and matrix [49,50].

At the same time, the increases in reinforcement contents also leaded to a linear increase of the Young’s modulus of the composites, an indicating of good reinforcement dispersion. The addition of natural fibers (stiffer phase) as reinforcement of a polymeric matrix (ductile phase) implies a resulting material with higher stiffness than the plain matrix. The deformation at rupture of the material decreases as the addition of reinforcement increases as a direct consequence of Hooke’s law.

**Figure 8:**
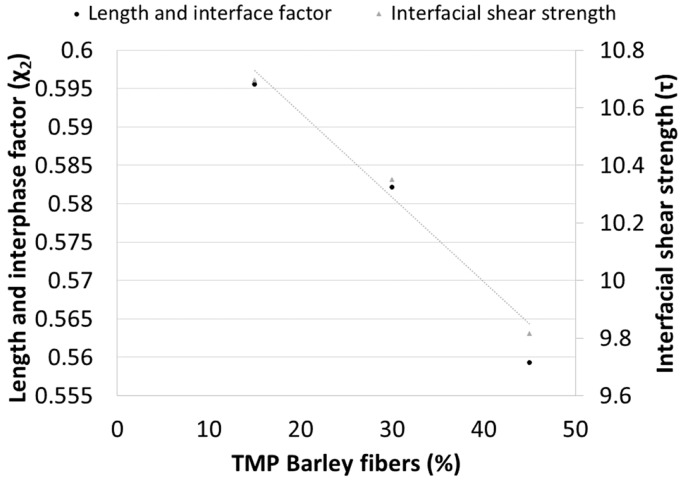
Evolution of the length and interphase factor and the interfacial shear strength as function of TMP barley fiber content.

The same micromechanics analysis on the length and orientation factor, and on the interfacial shear strength, was made to evaluate the impact of the reinforcement content over the same factors. The orientation factor was assumed to be 0.309 and the intrinsic tensile strength of the fibers 521.18 MPa. Figure 8 shows the evolution of both factors against the reinforcement content.

These two factors decrease almost linearly as the percentage of reinforcement increases. However, the interface strengths obtained between 15–45% reinforcement with the addition of a 6% coupling agent can be considered strong. In this case, both parameters evolved similarly, but a normalization of the scale of both parameters shows how the influence of the length and interface factor changes from positive for the composite adding 15 wt % of reinforcement to negative for the composite adding 45 wt %. This is explained by the decreases of the mean length of the fibers due to attrition phenomena during mixing. The mean length of the fibers decreased with the reinforcement content. Thus, longer fibers have more influence in the tensile strength of a composite than shorter ones. Nonetheless, the deviations of such parameter are slight and the main parameter commanding the tensile strength of the composites is the interfacial shear strength.

## 4. Conclusions

Thermomechanical barley fibers were used as reinforcement for a BioPE-based composite. A composite adding 30 wt % of reinforcement was used to evaluate the effect of a coupling agent over the tensile strength of the composites. It was found that a 6% MAPE content returned the highest tensile strengths. 

A micromechanics analysis allowed us to obtain the intrinsic tensile strength of the reinforcements, with a value of 521.18 MPa. This value is similar to other more commonly used natural reinforcements, ensuring the strengthening capabilities of the barley fibers. The analysis also allowed us to evaluate the strength of the interface. This interface was evaluated by the intrinsic tensile strength, with a value of 10.42 MPa, almost equal to von Mises criteria, and the coupling factor with a value of 0.18 inside the range from 0.18 to 0.20 considered optimum for semi-aligned short fiber–reinforced composites. The analysis also unveiled the higher impact of the strength of the interface over the morphology of the fibers for the contribution of the reinforcements to the tensile strength of a composite, when the percentage of fibers is constant and the percentage of coupling agent changes.

The effect of reinforcement content over tensile strength was also assessed. Composites adding 15 and 45 wt % of reinforcement were prepared and tensile tested. It was found that the tensile strength of the composites evolved linearly with reinforcement contents. It was found that the interfacial shear strength mainly commanded the contribution of the fibers to the tensile strength of the composites. Nonetheless, the length and interface factor also showed a slight effect, as a direct consequence of the shortening of the mean length of the fibers against fiber content.

## Figures and Tables

**Table 1:** Chemical composition of raw material, barley thermomechanical (TMP) fibers, and spruce TMP fibers.

	Holocellulose (%)	Klason Lignin (%)	Extractives (%)	Ash (%)	Length ^1^ (μm)	Diameter (μm)
Barley straw	70.12 ± 0.54	16.45 ± 0.34	5.90 ± 0.76	7.1 ± 0.2	–	–
Barley TMP fibers	77.67 ± 0.61	15.30 ± 0.46	2.73 ± 0.12	4.3 ± 0.3	745	19.6
Spruce TMP fibers	73.75 ± 0.83	25.80 ± 0.22	0.25 ± 0.34	0.2 ± 0.2	978	24.7

^1^ Length weighted in length.

**Table 2:** Tensile properties of barley TMP/ biopolyethylene (BioPE) composite with different coupling agent contents.

MAPE (%)	*V^F^*	$\sigma_{t}^{C}$ (MPa)	$E_{t}^{C}$ (GPa)	$\varepsilon_{max}$ (%)	$\sigma_{t}^{m^{*}}$ (MPa)
0	0	18.05 ± 0.74	1.06 ± 0.08	12.18 ± 0.34	18.05
0	0.233	18.82 ± 0.60	1.73 ± 0.10	2.88 ± 0.27	13.29
2		23.51 ± 0.39	1.76 ± 0.05	3.37 ± 0.15	14.19
4		29.84 ± 0.19	1.85 ± 0.07	5.19 ± 0.22	16.27
6		34.70 ± 0.90	2.14 ± 0.04	5.47 ± 0.31	16.44
8		32.65 ± 0.69	1.93 ± 0.05	5.67 ± 0.17	16.55

**Table 3:** Tensile properties of barley TMP/BioPE composites.

Barley TMP (%)	*V^F^*	$\sigma_{t}^{C}$ (MPa)	$E_{t}^{C}$ (GPa)	$\varepsilon_{max}$ (%)	$\sigma_{t}^{m^{*}}$ (MPa)
0	0	18.05 ± 0.74	1.06 ± 0.08	12.18 ± 0.34	18.05
15	0.111	25.2 ± 0.64	1.85 ± 0.06	7.65 ± 0.24	16.37
30	0.233	34.7 ± 0.90	2.59 ± 0.04	6.45 ± 0.31	16.76
45	0.367	43.1 ± 0.57	3.55 ± 0.05	4.69 ± 0.33	15.86
